# Combined surgical treatment and intraoperative adult stem cell application for osteochondral lesions of the knee: a case report and therapeutic approach

**DOI:** 10.1093/jscr/rjag365

**Published:** 2026-05-11

**Authors:** Eduardo Ciarrochi, Juan B Ciarrochi

**Affiliations:** Department of Regenerative Medicine, Clínica de Cirugía Especializada, Ciudad Autónoma de Buenos Aires, Argentina; Department of Regenerative Medicine, Clínica de Cirugía Especializada, Ciudad Autónoma de Buenos Aires, Argentina

**Keywords:** knee, osteochondral lesions, stem cells, orthopedics, immunomodulation

## Abstract

The knee is a complex joint that enables a wide range of movements. It plays a key role in load bearing and joint stability. The limited regenerative capacity of cartilage, coupled with the progression to disabling conditions such as osteoarthritis, underscores the need for early intervention in osteochondral lesions. In this context, stem cell therapy has emerged as a promising orthopedic tool due to its regenerative and immunomodulatory properties. We present the case of a patient with chronic osteochondral knee lesions who experienced significant impairment in quality of life due to pain, swelling, and joint locking. The patient underwent surgery combined with the intraoperative application of adult stem cells. During a 25-month follow-up period after surgery a remarkable clinical improvement was observed, including the recovery of joint function and sustained symptom relief. This combined approach supports a novel therapeutic strategy for the structural and functional regeneration of the knee joint.

## Introduction

The knee is a complex joint that enables a wide range of flexion, extension, and rotational movements, which confer its main function of maintaining body stability to bear loads. A disruption in this function can cause joint instability, a problem that affects both athletes and not athletes [1], thus predisposing them to both acute and chronic joint injuries due to changes in the load that must be borne by joint surfaces that are not prepared to withstand these stresses. The abnormal load increases friction and stress on the joint, causing damage to the collagen network within the extracellular matrix, increasing the production of catabolic factors such as metalloproteinases [2]. Articular cartilage defects have poor self-repair, which can lead to extremely limiting complications such as osteoarthritis [3]. The latter causes a decrease in quality of life, limiting daily activities, and mobility due to inflammatory symptoms such as pain and stiffness [4].

These premises require early and timely treatment of osteochondral lesions to avoid this outcome. Over the years, therapy with stem cells from different sources has been gaining prominence in the field of orthopedics, promoting a bioregenerative and preventive approach to medical conditions previously treated only with surgical or replacement strategies [5]. The combination of surgical treatment with cell therapy has been successfully implemented for knee conditions, with both structural and clinical improvements [3, 6]. One of the most relevant properties of stem cells is their immunoregulatory ability, producing immunoregulatory factors capable of reducing the inflammatory response and promoting tissue regeneration at the joint level [7].

The objective of this paper is to present the clinical advances observed over a period of 25 months after implementing the surgical approach combined with stem cell therapy from the superficial abdominal fascia in a patient with chronic osteochondral lesions of the right knee that greatly affected his quality of life.

## Case report

**Figure 1:**
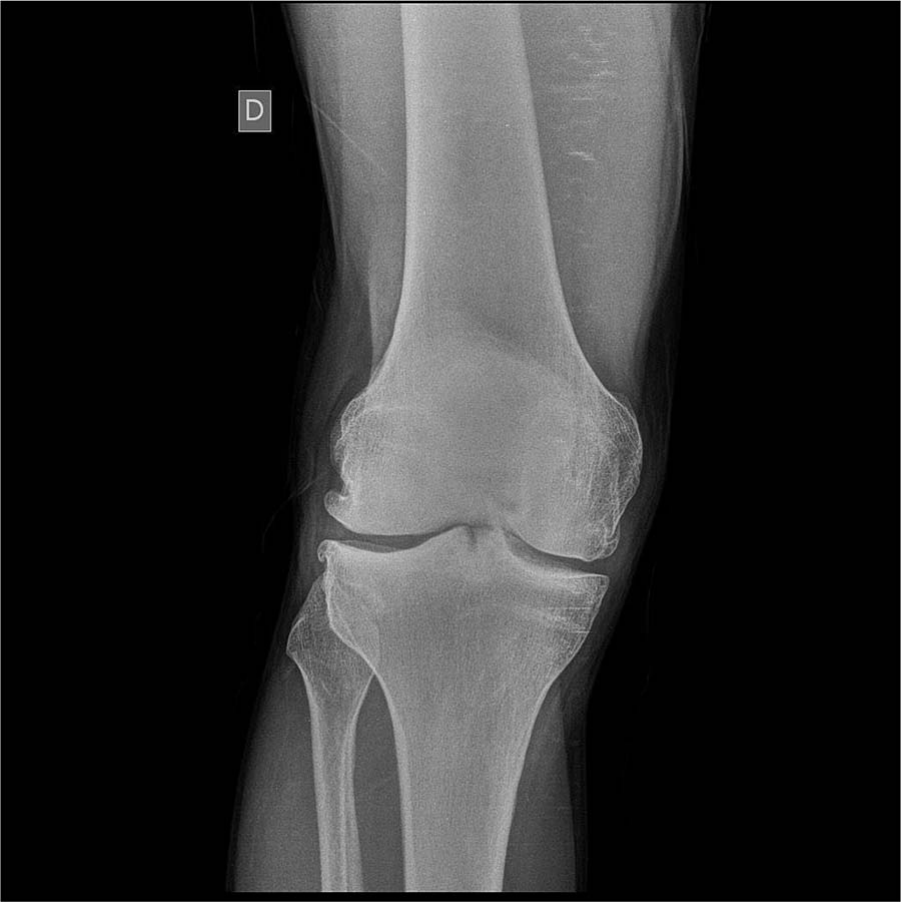
Frontal X-ray of the right knee showing joint space narrowing, osteophytes, and signs of subchondral sclerosis.

**Figure 2:**
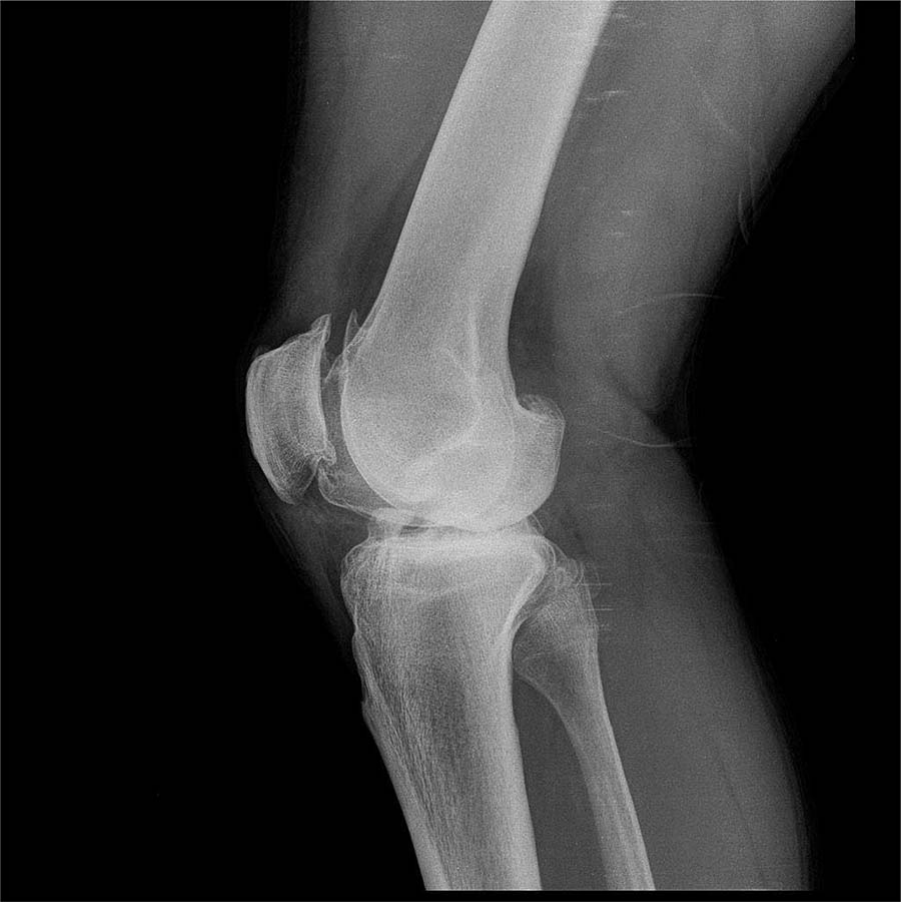
Lateral X-ray of the right knee showing marked joint involvement.

We present a 42-year-old patient with a history of surgical procedures on his right knee. His first surgery was performed after trauma to this knee at the age of 25. Since then, the functional ability of his joint has gradually declined. Over the years, a second unsatisfactory surgery was performed, and he was advised to stop his regular physical activities, thus reducing his quality of life. As a result, he developed a chronic osteochondral condition, which largely reduced his daily activities. Upon the first contact with the patient, knee joint assessment scales, such as the Tegner-Lysholm scale, Osteoarthritis Outcome Score (KOOS), and the EQ-5D visual analog scale, were performed, in which the patient reported going through one of his worst moments in terms of health. The interview and physical examination revealed that the cardinal symptoms experienced by the patient were instability, pain, swelling, and joint locking. All of this severely affected the patient's quality of life, preventing him from performing his daily sports practice. He even reported that he was already experiencing difficulties in daily activities such as getting up from his chair. Consultation included frontal and lateral X-rays of his right knee, which showed decreased joint space, marginal osteophytes, and signs of subchondral sclerosis (Figs 1 and 2). It was decided to perform surgical treatment combined with intraoperative stem cell application in December 2023. The patient agreed to the procedure.

In the days prior to surgery, autologous stem cells were harvested from the superficial abdominal fascia and cultured for subsequent intraoperative use. The superficial fascia was chosen as the source of stem cells due to the immunomodulatory properties of cells from this tissue [8]. A stem cell concentrate was prepared; no scaffold or collagen membrane was used.

**Figure 3:**
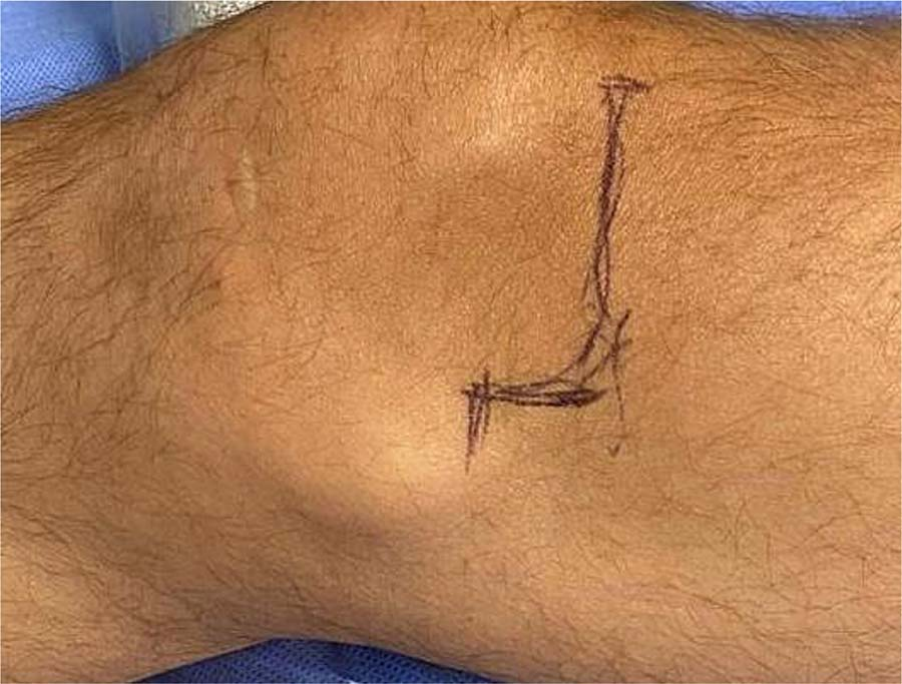
Incision marking.

On the day of surgery, an L-shaped incision was made in the anteromedial aspect of the knee (Fig. 3). The superficial planes were incised until the joint was reached. Intraoperative assessment confirmed the presence of chronic osteochondral lesion located in the weight- bearing area of the medial femoral condyle, consistent with preoperative images findings. The lesion was considered of moderate extent based on intraoperative evaluation and radiographic correlation. Identification and debridement of the lesion in the medial femoral condyle was carried out with adequate care to avoid damaging to the subchondral bone. Marginal osteophytes were meticulously removed. In addition, soft tissue adhesions were identified and removed (Video S1).

Subsequently, the stem cell concentrate was administered intra-articulary. The lesion was managed using a scaffold-free approach; therefore, no fixation or osteosynthesis was performed. Conventional closure of the skin was performed.

## Discussion

Stem cells are undifferentiated cells with the capacity for self-renewal and differentiation into various types of tissues. They interact closely with the immune system and can act on the inflammatory microenvironment present in various medical conditions [9]. In osteochondral lesions, changes in the loads that joint surfaces must bear generate abnormal mechanical stimuli that activate pathological and inflammatory factors, leading to joint atrophy and disease progression [10]. This generates an abnormal mechanotransduction at the joint level, which can be reverted with stem cell therapy [11].

These premises position stem cell therapy as an appropriate therapeutic tool in the field of orthopedics. Immediately after surgery, the patient was asked to perform flexion and extension movements, which he did successfully (Video S2). This was remarkable, taking into account that previously he had difficulty even getting up from his chair. Twenty-five months after the procedure, the patient's clinical improvement was evident, with decreased pain and joint locking, allowing him to resume his sports activities. The patient reports that his quality of life has completely changed and he is very satisfied with the procedure.

This case highlights surgical treatment combined with intraoperative application of adult stem cells as a valuable therapeutic approach for osteochondral lesions in orthopedics. Although further studies involving larger patient cohorts are required, this strategy demonstrates its potential to promote both structural and functional regeneration of the knee joint.

## Supplementary Material

rjag365_Supplemental_Files
